# The Advance of the Poor-Law Infirmary

**Published:** 1912-08-17

**Authors:** 


### THE ADVANCE OF THE POOR-LAW INFIRMARY.

### Dr. W. R. Jordan on the Value of a Visiting Staff.

Nothing in hospital progress during the past quarter of the nineteenth century was more remarkable than the development of the Poor-Law Infirmary, which the voluntary hospital spirit first permeated and then raised up into a friendly rival. Nowhere has this advance been more marked than in the great municipalities of the country, among which Birmingham has been a shining light in institutional as well as in municipal affairs. With the object, therefore, of illustrating this advance, our Commissioner recently visited Birmingham, and learnt from the lips of Dr. Walter Jordan, a visiting physician to the Birmingham Union Infirmary in Dudley Road, the chief steps in its development.

### The Beginning of Progress.

"The first of these," Dr. Jordan began, as we had traversed the outside of the great institution, "took place certainly in 1889, when the infirmary was first formally separated from the workhouse. In every instance this separation has been the essential precursor to infirmary progress, and it is to the credit of the Birmingham Guardians that they were one of the first parochial bodies to realise its importance and to welcome the change. It meant the appointment of a separate master and matron for the Infirmary, and by great good fortune the first matron was Miss Gibson, a lady who possessed not merely previous hospital experience, but a determined ambition to raise her department up to the best level of the voluntary hospital of the day. She has only recently retired after twenty years' invaluable work, for in her the medical staff found a most capable and enthusiastic ally in their attempts to make this infirmary the equal of the best hospitals in the City."

"What changes have taken place in the staffing of the infirmary?"

"At first there was a resident staff of four medical officers and a visiting staff of one surgeon and one physician. The visiting staff was, however, after the first year raised by the addition of two more physicians to its present total of four. Under the present system each visiting physician or surgeon has a resident assistant, and the infirmary is divided into four divisions, three medical and one surgical, each under the control of a member of the visiting staff."

### The Growth of Classification.

"Classification is general?"

"Yes; an infirmary with so large a number of patients as ours—namely, 1,300—makes that necessary, and we have in separate blocks, maternity, male and female epileptic, and an infectious diseases department. The work of the last of these has been affected by the extension of the City infectious diseases hospital. In addition, there is also a phthisis ward at the top of one of the pavilions. As you will have observed, the whole infirmary is built on the pavilion system, and the pavilions, each with three wards, radiate alternately from an immense corridor, which is nearly a quarter of a mile in length.

### The Infirmary Ward Unit.

Over one of these Dr. Jordan led the way, pointing out as he did so that the ward unit consisted of a linen cupboard, with a ward kitchen next door, and on the opposite side of the ward entrance a small ward containing two beds, which, however, is rarely in use. Each ward, except that devoted to the phthisical patients, contains twenty-eight beds; the windows rise to within a foot of the ceiling, this space being filled with an iron grating that ventilates to the outside air. At the far end of each ward is a balcony, wide enough to allow three beds to be wheeled upon it, with a brick parapet in front, roofed in, and the two ends terminated by brick walls, and entered from the ward by a door. On one side of this is the ward bathroom and basins, and on the other are water-closets, with a sink for cleansing bed-pans. Cross-ventilation between these and the ward is provided.

"The wards," Dr. Jordan remarked, "as you will see, are not uncheerful; the bricks are painted in two colours, and the ward floor is of oak. Flock pillows and mattress are provided."

"If the separation of the infirmary from the workhouse was the first step in the advance of the Poor-Law Infirmary, what do you consider the second?"

### The Importance of a Visiting Staff.

"The appointment of a visiting medical staff," replied Dr. Jordan without hesitation. "You see in many infirmaries the principal medical officer becomes inevitably the medical superintendent, and, human nature being what it is, there is a strong tendency for such an official to degenerate into a routineer. Some strong characters avoid this degeneration, and there are infirmary medical superintendents who need fear no comparisons, but it is useless to say that it does not occur. Beyond the Guardians, with whom naturally he wishes to work smoothly, there is no one to keep a resident superintendent in sole charge up to the mark. He is generally a full-time medical officer. He is not on the staff of a voluntary hospital. He is out of the world of friendly rivalry and hospital movement. Small wonder, then, if he grow to care little, so long as things jog on quietly. The atmosphere from which he is secluded is that in which are spent most of the working hours of a visiting staff. They, being on the staffs of hospitals themselves, import hospital demands, and are keen on the latest developments. They have better opportunity of knowing up-to-date medical requirements, and less inducement to refrain from pressing them on Guardians who, however energetic, are laymen. In my eight years' connection with the Birmingham Infirmary I have found the Guardians generally ready to accede to the visiting staff's demands, and I have found this also—where there is no visiting staff the resident assistants wait for promotion. Thus the institutional atmosphere tends to be handed on. Where there is a visiting staff the resident officers are usually junior men, who come, not for prospective appointments, but for experience. And this infusion of life and change makes for progress."

"The experience gained here is valuable?"

"I know none better for general practice. Our cases are those which the general practitioner is afterwards called on to treat. We see much of acute pneumonia, cardiac affections, nephritis, and those latest stages of nervous disease generally which are not seen in the voluntary hospitals. Cases of disseminated sclerosis and locomotor ataxia come and go in the general hospitals. They come to stay in the infirmaries. In surgery, too, our theatre offers much of value to the young practitioner. It is not my department, but I know that hysterectomy, hernia, and gall-bladder operations are frequently performed."

A Scarcity of Applicants.

"Posts here, then, are eagerly sought?"

"No; the infirmaries and the general hospitals are alike suffering from a dearth of applicants for resident posts. I attribute this to three causes. In the first place many more resident posts have been created than was formerly the case. Then, again, fewer men are entering the profession. I have not the statistics before me, but last year the number of entries into medicine was hardly more numerous than that of the preceding year. Then there is the competition created by such new posts as the school medical officer and what I may call the education service generally. Why, one large hospital in Birmingham has advertised for two months in vain. The Guardians here have consulted with me about it. A better salary is offered; the experience, as I have shown, is very good; and yet we have sometimes to fall back for considerable periods on *loca tenentes*. But, as I say, the voluntary hospitals are in the same position."

The Growth of Birmingham and Infirmary Progress.

"What effect on infirmary progress is the extension of Birmingham likely to have?"

"Ah! that is what everyone is asking. Its immediate effect is to give the Birmingham Board three infirmaries: the old Birmingham Infirmary, in which we are now talking, King's Norton, and Aston. An Infirmaries Committee has been appointed, with a sub-committee for each of the three. As to the effect of this, I can only say at present that it will mean generally a further extension of classification: and the extension of classification has been so far the formula of infirmary progress. All sorts of changes are talked of. A children's infirmary even is suggested, but that may or may not be formed. The phthisical patients, some suggest, will be moved to King's Norton, where the atmosphere is better. I have heard no definite suggestions in regard to Aston, but if any of these schemes, or others, are carried out, we may, perhaps, expect to receive a greater proportion of the acute cases here at Dudley Road."

The Latest Development.

"What has been the latest development?"

"In the past year we have not been idle. A new bacteriological and pathological laboratory has been built, and a whole-time bacteriologist has been appointed. We can thus undertake the absolutely up-to-date examination of all specimens, and manufacture our own vaccines. This progressive development was carried out on the proposal of Dr. Kauffmann, who was supported by the visiting staff, and the Committee, under the chairman, Mr. W. Darby, readily approved the proposal. So did the Local Government Board, after some delay. In addition, a new $x$-ray department has been built, though it has not yet been opened. By the way, I forgot to mention that in the last five years the phthisis cases have been separated from the patients in the general wards, and have now two wards to themselves, with an air-space per bed twice that of the ordinary wards."

"Then as to the children?"

"The idea of the Guardians has always been to have no workhouse children; and also they have interpreted destitution with some mercy, thus enabling some of its worst forms to be prevented by admitting patients before it was absolutely too late. These two progressive ideas have led to further classification, so that the older children are brought up at the Summerhill Home, and only babies are to be found in the workhouse."

From 1889 to 1912.

"I mention these facts to show how the sick wards which formed a mere adjunct to the workhouse before 1889 have developed into this large classified institution. So the new extension of the city, which now embraces practically all which was known as Greater Birmingham, has carried this process, after twenty-three years, one step further, and promises classification not by pavilions, but by a series of specialised institutions. Since the Poor-Law Infirmary was first recognised as an independent institution the growth of classification has marked the steady steps of its advance. Birmingham may be not a little proud of this; for as it is only the second city outside London which possesses an orthopædic hospital, so it was one of the first to foster Poor-Law Infirmary progress."

